# Evaluation of recombinant invasive, non-pathogenic *Eschericia coli *as a vaccine vector against the intracellular pathogen, *Brucella*

**DOI:** 10.1186/1476-8518-7-1

**Published:** 2009-01-06

**Authors:** Jerome S Harms, Marina A Durward, Diogo M Magnani, Gary A Splitter

**Affiliations:** 1 Department of Pathobiological Sciences, University of Wisconsin-Madison, 1656 Linden Drive, Madison, WI 53706, USA

## Abstract

**Background:**

There is no safe, effective human vaccine against brucellosis. Live attenuated *Brucella *strains are widely used to vaccinate animals. However these live *Brucella *vaccines can cause disease and are unsafe for humans. Killed *Brucella *or subunit vaccines are not effective in eliciting long term protection. In this study, we evaluate an approach using a live, non-pathogenic bacteria (*E. coli*) genetically engineered to mimic the brucellae pathway of infection and present antigens for an appropriate cytolitic T cell response.

**Methods:**

*E. coli *was modified to express invasin of *Yersinia *and listerialysin O (LLO) of *Listeria *to impart the necessary infectivity and antigen releasing traits of the intracellular pathogen, *Brucella*. This modified *E. coli *was considered our vaccine delivery system and was engineered to express Green Fluorescent Protein (GFP) or Brucella antigens for *in vitro *and *in vivo *immunological studies including cytokine profiling and cytotoxicity assays.

**Results:**

The *E. coli *vaccine vector was able to infect all cells tested and efficiently deliver therapeutics to the host cell. Using GFP as antigen, we demonstrate that the *E. coli *vaccine vector elicits a Th1 cytokine profile in both primary and secondary immune responses. Additionally, using this vector to deliver a *Brucella *antigen, we demonstrate the ability of the *E. coli *vaccine vector to induce specific Cytotoxic T Lymphocytes (CTLs).

**Conclusion:**

Protection against most intracellular bacterial pathogens can be obtained mostly through cell mediated immunity. Data presented here suggest modified *E. coli *can be used as a vaccine vector for delivery of antigens and therapeutics mimicking the infection of the pathogen and inducing cell mediated immunity to that pathogen.

## Background

There is no safe, effective human vaccine against brucellosis [1]. Brucellosis is a zoonotic disease causing chronic fatigue, arthritis, recurrent fever, endocarditis, and orchitis in humans [2,3]. The etiologic agents for brucellosis are the closely related, facultative, gram-negative, intracellular coccobacilli, *Brucella *species [4,5]. The ease with which *Brucella *can be transmitted by aerosolization, and the unpredictable timing of the onset of symptoms raise the specter of a potentially insidious bioterror attack [6-9]. During the course of infection, *Brucella *are actively phagocytosed by macrophages or other phagocytic cells where they prevent phagosome-lysosome fusion, persist and replicate in endocytic compartments that acquire endoplasmic reticulum membranes [10,11]. Bacteremia occurs during an acute phase that is hard to define or detect [12,13]. Live attenuated *Brucella *strains are widely used to vaccinate animals against brucellosis. However, these live *Brucella *vaccines can cause disease and are unsafe for humans [14-17]. Killed *Brucella *or subunit vaccines are not effective in eliciting long term protection [18]. Therefore, a new vaccine approach is needed.

Eliciting a specific T cell response is necessary to fight *Brucella *infection. Numerous studies have shown that Th1 or cell mediated immunity is crucial for protection against brucellosis [19] however Th2 or humoral immunity also participates in protecting the host [20-23]. Adoptive transfer of *Brucella *immune T cells protects mice against virulent *Brucella *infection [24,25] with both CD4^+ ^and CD8^+ ^T cells involved in immunity [26,27]. Nevertheless, murine brucellosis is markedly exacerbated in MHC I knockout mice that lack CD8^+ ^T cells compared to CD4^+ ^T cell deficient mice or wild type mice [19]. In fact numerous studies have shown that a CTL response is key to effective *Brucella *immunity [26,28-30].

Our approach utilizes a non-pathogenic *Escherichia coli *to mimic the intracellular pathogen *Brucella melitensis *in delivery and presentation of antigens to stimulate a Th1 and CTL response. *E. coli *are normally extracellular while *Brucella *are intracellular bacteria. Therefore, we engineered *E. coli *(DH5α) to express a plasmid containing the *inv *gene from *Yersinia pseudotuberculosis *and the *hly *gene from *Listeria monocytogenes *[31]. Introduction of *inv *confers *E. coli *invasion of host cells by binding the αβ1-integrin heterodimer. Upon clustering of integrins, invasin activates signaling cascades. One signaling pathway causes activation of components of focal adhesion complexes including *Src*, focal adhesion kinase, and cytoskeletal proteins, leading to the formation of pseudopods that engulf the bacteria into the host cell. Binding of invasin to β1-integrin is necessary and sufficient to induce phagocytosis of the bacteria even by non-professional phagocytic cells. A second pathway including activation of Rac1, NF-κB, and mitogen-activated protein kinase, leads to production of proinflammatory cytokines [32]. After internalization, *E. coli *is taken into the phagosome/lysosome where lysis of the bacterium occurs. The *hly *gene product, along with other bacterial proteins, is release into the lysosomal vesicle. The sulfhydryl-activated hly, also known as listeriolysin O (LLO) is a pore-forming cytolysin capable of binding and perforating phagosomal membranes at low pH [33]. The cytoplasmic contents of the bacteria can then escape into the cytosolic compartment of the mammalian cell through the pores generated by LLO. This critical step exports antigen from the *E. coli *into the cytosol where further processing by proteosomes and translocation by TAP into the endoplasmic reticulum lumen occurs for MHC class I presentation [34]. LLO is sufficient for MHC class I presentation of Ag when co-expressed in *E. coli *that are phagocytosed by Antigen Presenting Cells (APC) such as macrophages and dendritic cells [34,35]. Using similar recombinant *E. coli*, others have shown successful delivery of genes and molecules [31,34-44]. In this study, we investigate the potential of *inv-hly *expressing recombinant *E. coli *as a vaccine vector for immunization against the intracellular pathogen, *Brucella*.

## Methods

### Cell culture

Cells were maintained in RPMI 1640 medium (Invitrogen) supplemented with 10% fetal calf serum (FCS), 4.5% dextrose, 1 mM sodium pyruvate, and antibiotic-antimycotic solution (100 μ/ml penicillin G sodium, 100 μg/ml streptomycin sulfate, 0.25 μg/ml amphotericin B). In addition, drugs used for selection were: Blasticidin-S (Invivogen; 10 μg/ml) and G418-sulfate (Alexis Biochemical; 400 μg/ml). Cell lines included: D17 (ATCC CCL-183), TB1 (ATCC CCL-88), J774A.1 (ATCC TIB-67), HeLa S3 (ATCC CCL-2.2), RAW 264.7 (ATCC TIB-71), HEK 293 (ATCC CRL-1573), FLK [45], and the cytotoxicity target cell line RAW/YFP [45].

### Mouse care and vaccination

BALB/c female mice (H-2^d^), 4–6 wks old were purchased from Jackson Laboratory and injected with 0.1 ml of PBS i.p. one day prior to *E. coli *vaccinations to prevent the mice from succumbing to LPS-induced endotoxic shock from live *E.coli*. Intraperatoneal (i.p.) route of vaccination was chosen to best deliver live *E. coli *vector vaccine to mice based on consistency of results and ease of method. Recombinant *E. coli *vaccines were injected i.p. with 2 × 10^7 ^*E.coli *in PBS. PBS was used for negative controls. For experiments examining primary immune response cytokine profiles, mice were injected with *E. coli *vector vaccine and after 5 h, euthanized and spleens removed. For experiments enumerating antigen-specific CD8^+ ^T cells, RAW264.7 macrophages (H-2^d^) expressing GFP (RAW/GFP; [45]) was subjected to gamma-irradation (2 KR) and 1 × 10^6 ^cells in PBS were vaccinated in mice i.p. following the same protocol as the *E. coli *vaccines. Animals were boosted with the same dose two weeks later. Four weeks after the final boost, animals were euthanized and spleens harvested and processed for CTL assays. Live imaging was performed (IVIS; Caliper Biosciences, Inc.) with animals anesthetized using Isofluorane. IVIS image analysis was performed using Living Image 3.0 software (Caliper Biosciences). Each group of mice consisted of 4 animals. All animal experiments were conducted with approval from the Institutional Animal Care and Use Committee.

### Plasmid constructs

The prokaryotic expression vector pGB2Ωinv-hly [41](10.05 kb; spectinomycin resistance) was a gift from C. Grillot-Courvalin and expresses *invasin *from *Yersinia pseudotuberculosis *and *listeriolysin O *(LLO) from *Listeria monocytogenes*; pMC221 [46](4.9 kb; chloramphenicol resistance) expresses uvGFP; pXen-13 (pSK luxCDABE; 8.8 kb; ampicillin resistance) was obtained from Caliper Life Sciences and carries the *luxCDABE *operon for engineering bioluminescent Gram-negative bacteria. The eukaryotic expression vector pEYFP-N1 (4.7 kb; kanamycin resistance) was purchased from Clontech and expresses enhanced yellow fluorescent protein (EYFP); pORF-mIL12 (4.8 kb; ampicillin resistance) was purchased from Invivogen and expresses both chains of a functional murine IL-12 connected by a linker. The retroviral vector pLNCX2/EYFP [45](kanamycin/neomycin resistance) was engineered using pLNCX2 (BD Biosciences) and the EYFP from pEYFP-N1. The retroviral vector pLNCX2/BMEII1097 was engineered similarly using the *Brucella *BMEII1097 gene from pDONR201/BMEII1097 of the *Brucella *ORFeome purchased from OPEN Biosystems [47]. BMEII1097 is a probable transcription regulator *syr*B. This retroviral vector was used to transduce Raw 264.7 cells to be used as targets for CTL assays. The prokaryotic expression vector pDEST17/BMEII1097 was engineered from pDEST17 (invtrogen) and pDONR201/BMEII1097.

### *E. coli *vector vaccines

All *Escherichia coli *used in these studies were strain DH5α™ (Invitrogen) except for recombinants expressing pDEST17 vectors were we used BL21-AI™ (Invitrogen). Table 1 describes the recombinant *E. coli *vector vaccines.

**Table 1:** *E. coli *vector vaccines

**Name**	**Plamid(s)**
Invasive *E. coli*	pGB2Ω-inv-hly
*E. coli *gfp	pMC221
*E. coli *gfp+inv	pMC221, pGB2Ω-inv-hly
*E. coli *gfp+inv+IL12	pMC221, pGB2Ω-inv-hly, pORF-mIL12
*E. coli *inv+IL12	pGB2Ω-inv-hly, pORF-mIL12
Bioluminescent non-invasive *E. coli*	pXen13™
Bioluminescent invasive *E. coli*	pXen13™, pGB2Ω-inv-hly
*E. coli *EYFP	pEYFP-N1
*E. coli *inv EYFP	pEYFP-N1, pGB2Ω-inv-hly
*E. coli *inv B7^a^	pGB2Ω-inv-hly, pDEST17-BmeII1097

^a^All *E. coli *used in these studies were DH5a™ except *E. coli *inv B7 which is strain BL21-AI™.

### Invasion and gene delivery assays

One day prior to cell infection, eukaryotic cell lines were seeded at 2 × 10^5 ^cells/well in a six-well plate (or two well chambered coverslips for fluorescent microscopy) in 2 ml/well RPMI with 10% fetal calf serum (Invitrogen) and grown in a humidified CO_2 _incubator at 37°C. *E. coli *were grown overnight in a shaking incubator at 37°C in LB broth (Difco) supplemented with appropriate antibiotic for plasmid selection. The following day, bacteria were counted by 600 nm absorbance spectrometry and added to washed eukaryotic cells in fresh medium without antibiotic at the specified MOI. Bacteria were then centrifuged onto the monolayer at 2 krpm for 5 min at room temperature. Cells were incubated for 90 min, washed and fresh medium added supplemented with 100 μg/ml gentamicin to kill extracellular bacteria. For invasion assays, cells were incubated for an additional 90 min to kill extracellular bacteria, then washed and lysed in 200 μl of 1% triton X-100 for 5 min at room temperature. Finally, 800 μl of LB broth was added to each well and CFU were determined on LB agar plates supplemented with chloramphenicol, the selection drug for the GFP plasmid. For gene delivery assays, cells were incubated then analyzed by fluorescent microscopy. Random fields of cells were counted and scored for fluorescence at indicated times. For IL-12 assays, infected cells were fixed and permeabilized using Cytofix/Cytoperm™ (BD Biosciences) following the manufacturer's protocol. Samples were stained using IL-12 (p40/p70) PE conjugated monoclonal antibody (BD Biosciences) and analyzed by flow cytometry.

### MHC class I pentamer staining and cytokine profiling

Pooled splenocytes from four mice per immunization group were isolated and density gradient purified (Fico/Lite-LM (Mouse); Atlanta Biologicals). Leukocytes were subjected to non-T cell depletion using a Pan T Cell Isolation Kit and MACS separation (Miltenyi Biotec) following the manufacturer's protocol. Aliquots of 2 × 10^6 ^T cells were then used for flow cytometry or cytokine profiling. R-PE labeled Pro5^® ^MHC class I pentamers GFP antigen specific for T cell receptors of H-2K^d ^HYLSTQSAL were co-stained with FITC labeled rat anti-mouse CD8α and used for flow cytometry along with controls following the manufacturer's suggested protocol (Proimmune). Controls included R-PE labeled rat anti-mouse CD3ε (SouthernBiotech), and R-PE and FITC anti-rat IgG2a and anti-rat IgGκ (BD Biosciences). Flow cytometry analysis was performed on 3.5 × 10^5 ^cells for each immunization group. For cytokine profiling, T cells from immunized and control mice were incubated with gamma-irradiated (2 KR) RAW 264.7 macrophages on 6 well plates with or without the addition of 50 mM GFP peptide (HYLSTQSAL; A&A Labs LLC) for 3 days. Supernatant was harvested, centrifuged to remove cell debris and processed using a Th1/Th2 cytokine kit by cytometric bead array (BD Biosciences). Data acquisition and analysis was performed according to the manufacturer's instructions using flow cytometry.

### Cell mediated cytotoxicity

Splenocytes from immunized mice were isolated and gradient purified (described above) for use as effector cells. Transduced RAW 264.7 cells expressing GFP or BMEII1097 were cloned by limiting dilution and used as target cells. Cytotoxic effector cells were expanded *in vitro *by growth on confluent 2 KR gamma-irradiated target cells in six-well plates supplemented with 10% T-stim without Con A (BD Biosciences) for three days. Effector cells were then washed and purified through a density gradient. Cells were counted and assayed using a CytoTox 96^® ^Non-Radioactive Cytotoxicity kit (Promega) following the manufacturer's protocol with 4 h incubation.

### Flow cytometry

Acquisition was performed on a FACSCalibur flow cytometer (BD Biosciences) and analyzed using FlowJo 8.7.1 software (Tree Star, Inc).

### Cell transfection and transduction

Retrovirus-mediated gene transfer was accomplished using the BD Retro-X System (BD Biosciences) following the manufacturer's suggested protocol. Briefly, 100 × 20 mm tissue culture dishes (Falcon) were seeded with the packaging cell line GP2-293 at 70–90% confluency. GP2-293 cells were co-transfected with 5 μg each of retroviral vector and the envelope glycoprotein expression vector pVSV-G using 15 μl/transfection of Lipofectamine 2000 (Invitrogen) for 3 h in a total volume of 5 ml medium/dish. Subsequently, transfection medium was replaced with 10 ml growth medium, and the cells incubated for 72 h. Retrovirus-containing supernatant was harvested, filtered (0.45 μm), and concentrated by ultracentrifugation. Supernatant was removed and virus resuspended in the residue (~200 μl) and frozen (-80°C). Cells for transduction were plated on 6-well tissue culture plates (Falcon) at 50% confluency. Concentrated retrovirus (titer unknown) along with polybrene (8 μg/ml) were added to 1 ml/well cells and incubated overnight. Transduction medium was replaced with fresh growth medium, and the following day cells were split into appropriate selective medium.

### Electron microscopy

Cell lines (2 × 10^5 ^cells/well) were incubated on glass coverslips in six-well plates overnight at 37°C in a CO_2 _humidified incubator. Using conditions as with invasion assays, invasive or non-invasive *E. coli *were incubated with the cells at MOI 100 for 90 min. The cells were thoroughly washed to remove extracellular bacteria followed by gentimycin incubation for an additional 90 min. Cells were washed in PBS and fixed in Karnovsky's Fixative (Electron Microscopy Sciences) following manufacturer's protocol. TEM was performed at the University of Wisconsin Medical School Electron Microscope Facility . Figures were imported using Adobe Photoshop CS3 10.0.1.

### Statistical analysis

Student's t-test was performed and results expressed as the arithmetic mean with the variance of the mean (mean ± SE).

## Results

### The recombinant *E. coli *vaccine vector efficiently infects cells

**Figure 1:**
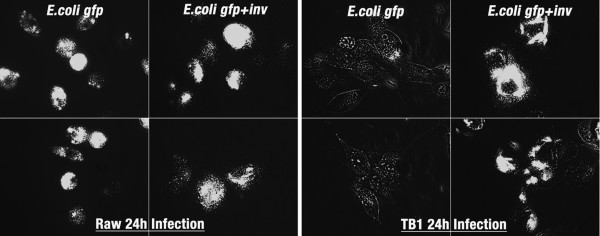
**Recombinant invasive *E. coli *infects phagocytic and non-phagocytic cells**. The macrophage cell line, RAW 264.7 and epithelial cell line, TB1, were incubated with GFP-expressing *E.coli *(*E. coli gfp*) or co-expressing invasin (*E. coli gfp+inv*) at MOI of 10 for 3 hours, washed, and after 24 h in gentimicin media, imaged by fluorescent microscopy. The image shows two representative fields at equivalent scale of each treatment and cell line.

**Figure 2:**
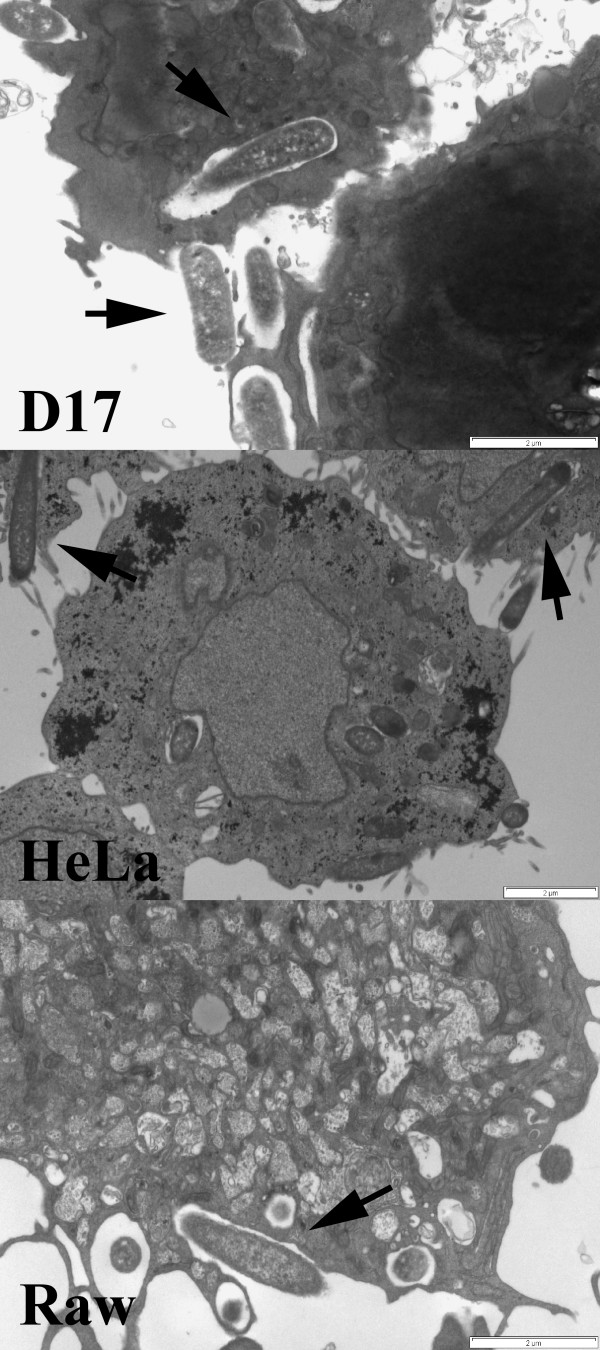
**Transmission Electron Microscopy shows recombinant invasive *E. coli *similarly engulfed by non-professional phagocytic cells (D17, HeLa) and phagocytic cells (Raw)**. The osteosarcoma cell line D17, epithelial cell line HeLa, and macrophage cell line Raw were incubated with recombinant invasive *E. coli *(MOI 10) for 3 hours, washed, fixed and processed for TEM. Image demonstrates each cell line engulfing *E. coli *(arrows) with lamellipodia. Scale bar indicates 2 microns.

The objective of this study was to take a non-pathogenic organism such as *Escherichia coli *and genetically engineer it to mimic infectivity and intracellular antigen trafficking of a pathogen such as *Brucella melitensis*. The engineered bacteria would then be employed as a vaccine vector for *Brucella *antigen delivery and evaluated for immune response. *E. coli *are normally extracellular, and taken up and destroyed by phagocytic cells such as macrophages. We transformed GFP expressing *E. coli *DH5α (*E. coli gfp*) with a plasmid encoding invasin from *Yersinia pseudotuberculosis *and LLO from *Listeria monocytogenes *(*E. coli gfp+inv*) and tested whether these *E. coli *were invasive to non-professional as well as professional phagocytic cell lines. Non-invasive *E. coli *(*E. coli-gfp*) or invasive *E. coli *(*E. coli gfp+inv*) were added to different cell lines and analyzed by fluorescent microscopy. Addition of invasive *E. coli *to all cell lines, phagocytic and non-phagocytic, resulted in intracellular fluorescent bacteria. However, only minimal non-invasive *E. coli *fluorescence was observed in non-phagocytic cell lines (D17, FLK, 293, TB1), but was present in macrophage cell lines (RAW and J774). An example with TB1 and RAW264.7 cells is shown in Figure 1. To further determine whether the invasive *E. coli *were intracellular, invasion assays were performed (Table 2). Note non-invasive *E. coli *were not recovered unless a high MOI was used. In contrast, large numbers of invasive *E. coli *were recovered from all cell lines analyzed. Furthermore, electron microscopy showed invasive *E. coli *bound to the cell surface and engulfed by lamellipodia consistent with invasin-integrin interactions (Figure 2). Non-invasive *E. coli *were also used in the TEM assay, but could not be detected within or surface-bound to any non-phagocytic cell line (data not shown).

**Table 2:** Intracellular bacterial survival (× 10^4^) per 2 × 10^5 ^eukaryotic cells

Cell line (*Macrophages)	MOI 10 (1.5 h Infection)		MOI 100 (1.5 h Infection)	
	
	*E.coli gfp*	*E.coli gfp+inv*	*E.coli gfp*	*E.coli gfp+inv*
D17	0	20	0	320
FLK	0	83	2	168
HEK293	0	46	15	627
TB1	0	2	4	345
J774*	0	18	19	270
RAW*	0	17	11	317

**Figure 3:**
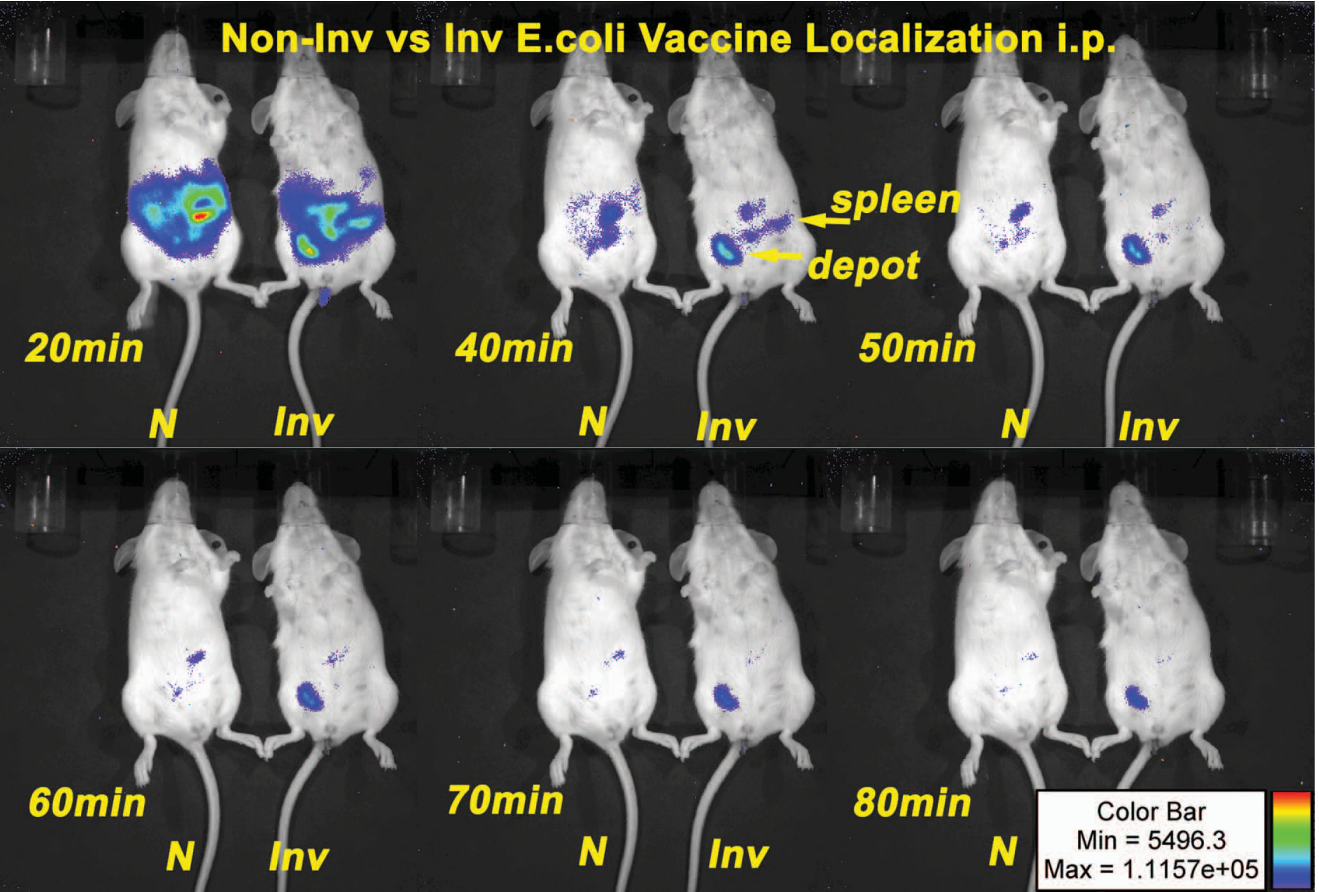
***In Vivo *biophotonic imaging of mice vaccinated with non-invasive (*N*) or invasive (*Inv*) bioluminescent *E. coli *indicate similar trafficking from the intraperitoneal site of injection but prolonged antigen expression of the recombinant invasive *E. coli *vaccine**. Mice vaccinated i.p. were anesthetized and imaged at time points indicated. After 80 min, bioluminescent invasive *E. coli *were still detectable at the site of injection indicating live bacteria.

Since our intent is to use the invasive *E.coli *as a live vaccine vector, we examined localization and persistence of the vector *in vivo*. We transformed *lux *operon expressing *E. coli *DH5α (constitutively bioluminescent) with the *inv-hly *encoding plasmid as our invasive *E. coli *(inv *E. coli*). Mice were intraperitoneal injected with non-invasive or invasive bioluminescent *E. coli *and analyzed by biophotonic imaging over time. Both bioluminescent species trafficked to the spleen. However, the invasive *E. coli *vector persisted longer at the site of injection suggesting an extended period of antigen delivery (Figure 3).

### The recombinant *E. coli *vaccine vector efficiently delivers therapeutics

Unlike *Escherichia*, *Brucella*, after being engulfed by the cell, escape phagosome lysis and multiply at the endoplasmic reticulum. Most likely, this process leads to MHC class I presentation of *Brucella *antigens by the host cell [48]. *Escherichia*, in contrast, are phagocytosed and rapidly destroyed with antigens being presented by MHC class II [49,50]. Therefore, the *inv *expressing plasmid co-expresses *hly *(hemolysin) to enhance MHC class I presentation of antigens carried by the invasive *E. coli *vaccine vector. Hemolysin (hly) or LLO perforates phagosomal membranes at low pH and the contents of the vaccine are released into the cytosol of the cell [51]. To test the functionality of the *hly *gene product in the *E. coli *vector, we first examined delivery of a eukaryotic expression plasmid, pEYFP-N1 expressing yellow fluorescent protein (YFP) under control of the eukaryotic CMV promoter, using fluorescent microscopy. Table 3 shows results after two or seven days post infection (MOI 100) of confluent cells lines. Only the LLO expressing *E. coli *vector transferred functional YFP plasmid to all mammalian cells tested. Interestingly, the number of YFP positive cells per total cells increased as time progressed. Also, two days post-infection no YFP positive macrophages (RAW, J774) were observed, but after seven days fluorescent positive cells were similar to the non-phagocytic cell lines.

**Table 3:** YFP gene delivery for mammalian cell expression (Fluorescent cells/10^3 ^total cells)

Cell line (*Macrophages)	*E.coli *[pEYFP-N1] (MOI 100)		*Inv E.coli *[pEYFP-N1] (MOI 100)	
	
	2 Days	7 Days	2 Days	7 Days
D17	2	0	67	450
FLK	4	0	74	300
HEK293	4	0	72	360
TB1	0	0	46	150
J774*	0	0	0	400
RAW*	0	0	0	500

**Figure 4:**
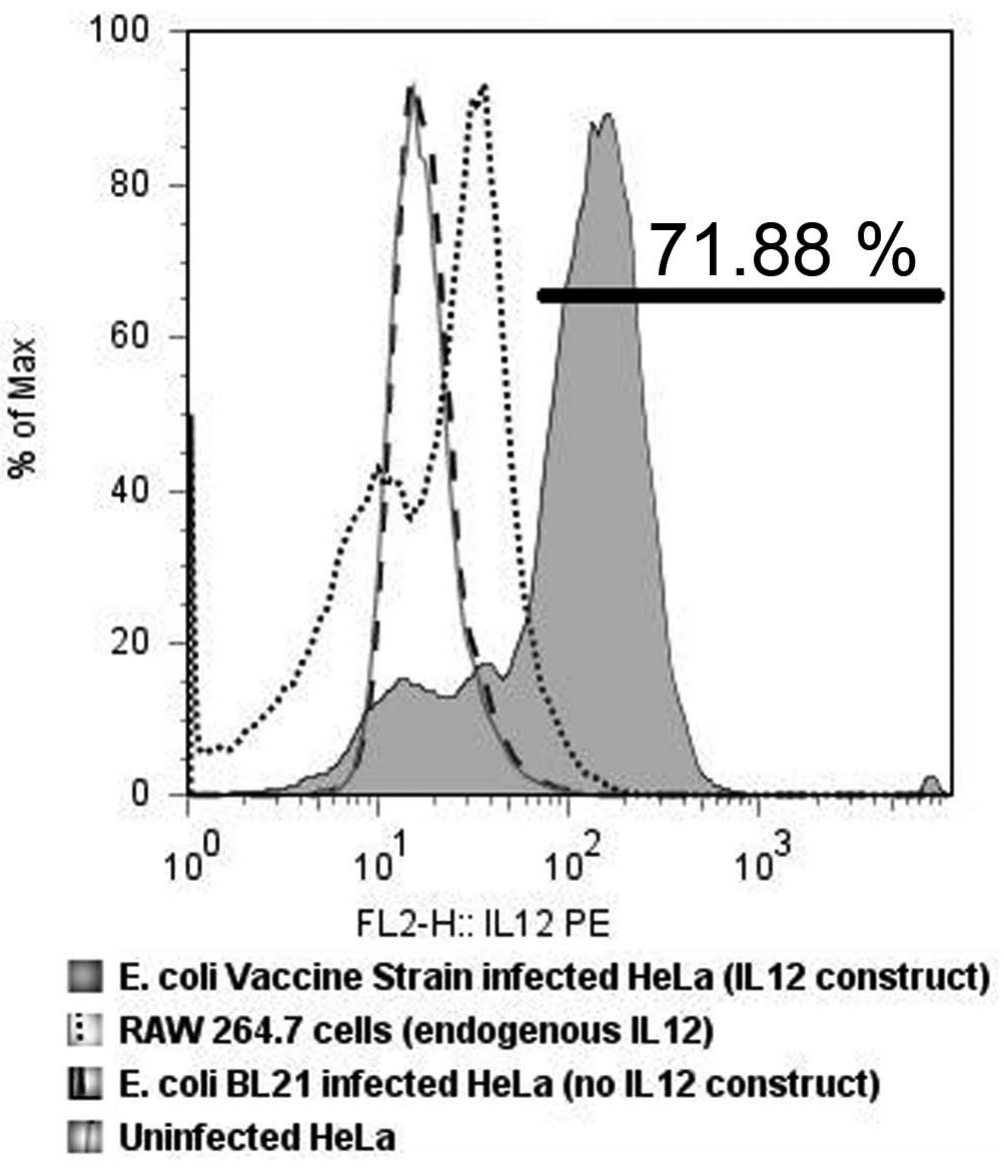
**Microfluorimetry of supernatant of HeLa cells expressing murine IL12 indicate efficient plasmid delivery after infection by recombinant invasive *E. coli *vaccine**. FACS analysis showed greater than 70% of HeLa cells were expressing murine specific IL12 at 72 h after 3 h infection with the invasive *E. coli *vaccine. The positive control was endogenous IL12 produced by the mouse macrophage cell line RAW 264.7. Negative controls included invasive *E.coli *not carrying the murine IL12 expression vector (*E. coli *BL21 infected HeLa supernatant) and uninfected HeLa cells supernatant.

Data indicate that the early choice of a Th1 (cellular) or a Th2 (humoral) immune response is dependent mainly on the balance between interleukin-12 (IL12), favoring a Th1 response, and interleukin-4 (IL4), favoring a Th2 response [52,53]. Vaccine studies have demonstrated that co-deliverance of IL12 with the antigen increases Th1 response to the vaccine [54-57]. Thus, we included a murine IL12 eukaryotic expression plasmid in the invasive *E. coli *vaccine vector and tested for delivery and expression of IL12 in cell culture. Using human HeLa cells, microfluorimetry analysis demonstrated greater than 70% of *E. coli *vaccine infected cells were positive for murine IL12 (Figure 4). This compared favorably to endogenous murine IL12 production by mouse Raw264.7 macrophage cell positive control. Therefore, the *E. coli *vaccine vector was effective in delivering therapeutics to the host.

### The recombinant *E. coli *vaccine vector induces a Th1 response

Since we were interested in preparing a vaccine that would stimulate cell mediated immunity, we analyzed for a Th1 cytokine profile and specific CD8^+ ^T cells. Performing real-time PCR gene expression profiling analysis on splenocytes from mice 5 h following vaccination with invasive *E. coli *vaccine or non-invasive *E. coli*, we analyzed for differences in primary immune response profiles. This time-point was chosen because typically, cytokines that promote T cell responses are measured 5 h post-immunization [58]. Table 4 lists fold gene expression from splenocytes of animals receiving recombinant *E. coli *vaccine compared to control *E. coli*. The data were difficult to interpret since both key Th1 and Th2 cytokines were upregulated in *E. coli *vaccine immunized animals compared to *E. coli *control immunized animals. Most likely, the complexity of the cytokine profile can be attributed to the highly stimulatory LPS of E. coli [58,59]. Comparison profiles of *E.coli *vaccinated animals to PBS control animals were also performed (data not shown), but the results were not relevant to our objective of determining whether the recombinant *E. coli *vaccine would elicit a different cytokine profile relative to control *E. coli*.

**Table 4:** Immune response gene profile of splenocytes after 5 h immunization with *E. coli *vaccine.

	Gene	Fold Regulation^a^		Gene	Fold Regulation
Th1 Regulation	*Csf2*	2.07	Th2 Regulation Genes	*Il10*	1.37
	*Ifna4*	5.10		*Il13*	3.14
	*Ifng*	1.11		*Il1f5*	-10.20
	*Il12b*	2.38		*Il4*	1.04
	*Il16*	6.73		*Il9*	9.25
	*Il18*	-1.04			
			
	*Il2*	1.27	Other Immune Response Genes	*Ifnb1*	-3.36
	*Il27*	-1.11		*Il1f10*	6.73
	*Tnf*	3.14		*Il1rn*	4.44
	*Tnfsf15*	11.71		*Il21*	6.28
	*Tnfsf4*	5.10		*Tnfsf8*	10.93
	*Cd40lg*	4.44		*Tnfsf9*	6.28

^a^Values are real-time PCR expression of recombinant invasive *E. coli *vaccinated animals relative to expression of non-invasive *E. coli *vaccinated animals. Data were compiled from triplicate wells. T-test p value < 0.001.

However, because of the mixed Th1/Th2 cytokine profile of the primary immune response, we decided to investigate whether the secondary immune response would give a more defining Th1 cytokine profile response to the antigen. RAW 264.7 macrophages were co-cultured with splenic T cells from groups of mice that had been immunized 4 weeks. Half of the cultures were supplemented with the H-2K^d^-binding peptide HYLSTQSAL of GFP and supernatants were measured for cytokines after three days. GFP nonamer treated cultures showed a large increase in Th1 cytokine levels in *E. coli *vaccine immunized T cell groups with negligible change or decrease in Th2 cytokine levels (Table 5). Production of IFNγ significantly increased for the two specific invasive *E. coli *vaccines, *GFPinv *and *GFPinvIL12 *whereas production of IL4 increased for the negative control vaccines, *GFP *and *()invIL12 *as well as significantly increasing in the *PBS *control samples. Although the primary response indicated a mixed Th1/Th2 profile, the secondary immune response indicates a shift to the Th1 profile. Identification of antigen specific CD8^+ ^T cells would confirm a Th1 profile and generation of cell-mediated immunity.

**Table 5:** Three day cytokine production (pg/ml)^a ^of vaccinated mouse splenic T cells cultured in macrophages with (+) or without (-) 50 μM GFP peptide HYLSTQSAL.

*Vaccine Group*	*Th1 Cytokines*					*Th2 Cytokines*				
	TNFα		IFNγ		IL2		IL4		IL5	
	
	(-)	(+)	(-)	(+)	(-)	(+)	(-)	(+)	(-)	(+)
***GFP***	43.3	**179.1**	421.6	520.8	0	**24**	3.2	5.2	51.9	*41.5*
***GFPinv***	90.7	**348.8**	628.9	**1353.7**	1.9	**32.2**	4.0	*3.8*	56.2	***10.2***
***GFPinvIL12***	79.5	**443.7**	349.3	**1307.2**	22.3	36.7	3.2	***0***	17.9	***5.8***
***()invIL12***	110.8	147.5	673.6	862.9	24.8	***3.1***	3.0	5.9	71.1	*41.4*
***RAW/GFP***	134.7	**566.2**	NA^b^	NA	10.4	**24.8**	11.6	***0***	14.4	***0***
***PBS***	127.2	*84.5*	130.0	**440.0**	2.2	3	0	**12**	0	**14.1**

^a^Values are from pooled T cells from four mice of each vaccine group. Data are compiled from two experiments. A significant change in expression is indicated by 2-fold or greater over non-peptide treated samples (p < 0.05) and is shown in **bold **text. Decreases are in *italics*.

^b^Not enough cells to perform assay.

**Figure 5:**
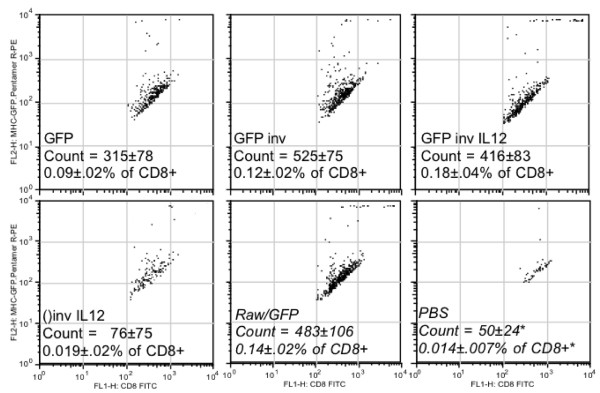
**FACS analysis of splenic T cells co-stained with anti-CD8 and H-2K^d^-GFP peptide pentamer indicate increased numbers of antigen specific CTLs in recombinant invasive *E. coli *vaccine immunized animals**. Groups of four mice were vaccinated with GFP-expressing *E.coli *that were either non-invasive (*GFP*), recombinant invasive (*GFP inv*), or recombinant invasive with murine IL12 expression vector (*GFP inv IL12*). Negative controls included recombinant invasive *E. coli *with the murine IL12 expression vector but without the GFP antigen (*()inv IL12*), and *PBS*. Positive control vaccine was irradiated mouse macrophage RAW cell line (H-2^d ^haplotype) constitutively expressing GFP (*Raw/GFP*). Vaccinated mice were boosted after two weeks, and splenocytes harvested after four weeks.

To determine the proportion of CD8^+ ^T cells specific for GFP antigen in the spleens of *E. coli *vaccine immunized BALB/c mice, we used H-2K^d ^MHC class I pentamer complex combined with the GFP peptide HYLSTQSAL (designated MHC-GFP pentamer) co-stained with CD8^+ ^antibody and analyzed by flow cytometry. As shown in Figure 5, the invasive *E. coli *vaccine induced GFP peptide specific CD8^+ ^T cells at a significant level (p < 0.05) greater than the non-vaccinated (*PBS*) and empty vaccine (*()inv IL12; *invasive without GFP) controls and at similar levels to mice given syngeneic APC's constitutively expressing the antigen (*RAW/GFP*). However, the non-invasive *E. coli *vaccine control (*GFP*) also induced notable levels of CD8^+ ^T cells not significantly different than the vaccines (*GFP inv *and *GFP inv IL12*). The high number of specific CD8^+ ^T cells induced by the invasive *E. coli *vaccines correlated with the Th1 cytokine up-regulation induced in the secondary immune response by these cells *in vitro *(Table 5). As a confirmation of *E. coli *vaccine generated cell mediated immunity, we analyzed cytolytic T lymphocyte (CTL) response.

### The recombinant *E. coli *vaccine vector induces specific CTL responses

**Figure 6:**
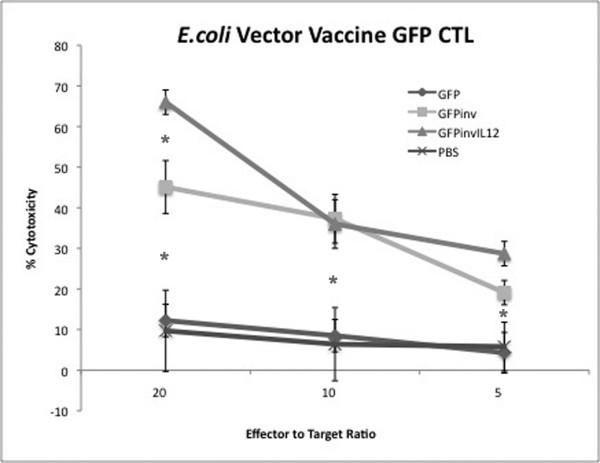
**Recombinant *E. coli *vaccine vector delivering GFP antigen induced higher CTL response**. Effector splenocytes of mice immunized with *E. coli*-GFP (*GFP*), our recombinant vaccine vector *E. coli*-GFP expressing invasin and hly (*GFPinv*), the recombinant vaccine vector also carrying the eukaryotic muIL12 vector (*GFPinvIL12*), or diluent control (*PBS*) were incubated with Raw/GFP target cells and assayed for cytotoxicity. Error bars represent quadruplicate wells. *GFPinvIL12 generated T cytotoxicity was significantly greater than *GFP *or *PBS *controls (p < 0.05).

Splenocytes of mice immunized with the invasive *E. coli *vaccine vector expressing the GFP antigen were used as effector cells in cytotoxicity assays against RAW/GFP target cell lines. As shown in Figure 6, the invasive *E. coli *vaccine vectors (*GFPinv*, *GFPinvIL12*) elicited marked CTL response against the target cells versus the control non-invasive *E.coli *(*GFP*) and mock immunized (*PBS*) mice. To optimize the immunization protocol, we repeated this experiment with mice vaccinated with different doses of *E. coli *vaccine ranging from 10^4 ^to 10^8 ^cells in both primary and booster vaccines. Results (not shown) demonstrated that the highest vaccine dose (10^8^) elicited the highest CTL results.

**Figure 7:**
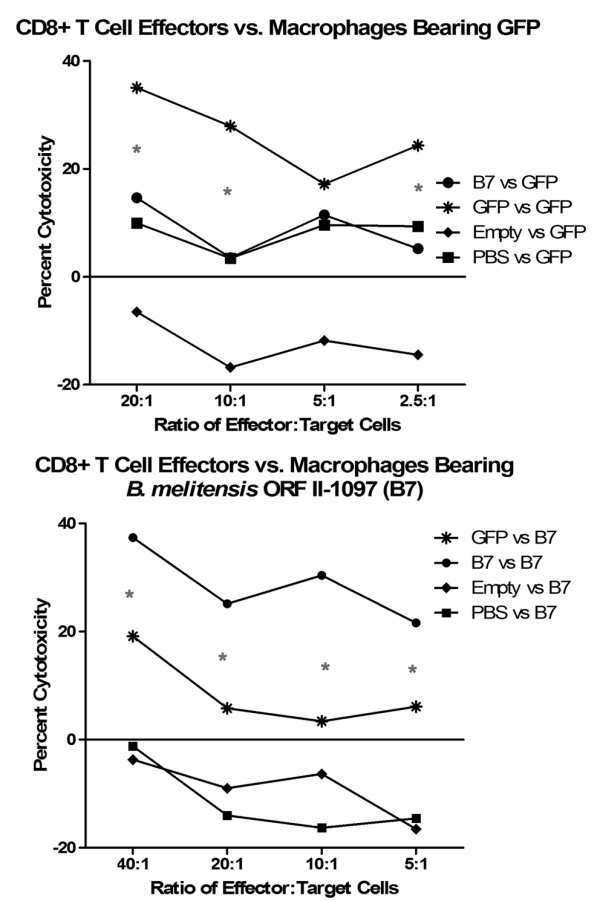
**Recombinant invasive *E. coli *vaccine vector induces specific CTL response**. *Inv-hly E.coli *vaccine vectors expressing *B. mel *ORF BmeII-1097 antigen (*B7*), GFP antigen (*GFP*), or no antigen (*Empty*), were used to immunize mice along with a negative (*PBS*) control. Splenocytes were isolated and used against target RAW macrophages expressing either GFP (*Raw/GFP*) or B7 (*Raw/B7*). Data demonstrate that CTLs generated by the *E. coli *vaccine were specific to antigen expressed by the vaccine. *GFP vs GFP and *B7 vs B7 *specific cytotoxicity were significantly greater (p < 0.05) than non-specific controls.

To identify the specificity of the CTL response, an *E. coli *vaccine expressing *B. melitensis *ORF BmeII-1097 (designated *B7*) as well as vaccine vector without antigen expression (designated *Empty*) was included. Antigen of this *Brucella *ORF had been determined by RANKPEP computer algorithm [60] to have high binding to mouse H-2K^d^. BmeII-1097 is a putative transcriptional regulator with homology to *syr*B. Cytotoxicity assays affirmed that CTLs generated by the invasive *E. coli *vaccine were specific to the expressed antigen of the vector (Figure 7).

## Discussion

There is no safe, effective vaccine against human brucellosis. The ability of *Brucella *to chronically infect humans is related to its ability to avoid a protective Th1 response [61-64]. Chronic brucellosis patients display a Th2 immune response [64,65]. Our objective was to analyze a novel vaccine approach engineering *E.coli *to mimic invasion, immunoregulation, and antigen expression of *Brucella *without the pathogenicity of *Brucella*.

Recombinant invasive *E. coli *have been used to deliver therapeutically relevant molecules to mouse and human professional and non-professional phagocytic cells [38,66-70]. To date, use of recombinant *E. coli *as vectors has mainly been for delivering DNA for genetic vaccination. The ability to easily be engulfed by cells in addition to the absence of plasmid size restrictions make bacteria an interesting vector for gene therapy. In most cases, the recombinant invasive *E. coli *is used to efficiently enter eukaryotic cells where it is destroyed, releasing a eukaryotic vector to the host cell for expression of a therapeutic gene [66]. Using this basic approach, we modified *E. coli *to be a live vaccine that would efficiently invade host cells, deliver a eukaryotic gene expression vector to help modulate the proper immune response, and release a large amount of antigen efficiently produced by the prokaryotic expression system. *E. coli *infection would not be long-lived, unlike live *Brucella*, being cleared by the host relatively rapidly. Nevertheless, we found our invasive *E. coli *could survive in host cells up to 72 h after infection compared to control *E. coli *surviving less than 3 h post-infection (data not shown). These data had been confirmed by others [41] and suggest an alternate pathway of infection for our recombinant vaccine *E. coli*.

Bacteria enter cells through a variety of receptors. Host cell receptor(s) for binding and internalization of *Brucella *have not been identified but involve lipid rafts and components of this micro domain [71]. The *Brucella *endocytic pathway is distinct from the classical endosome-lysosome pathway in that *Brucella *inhibit phagosome-lysosome fusion [10]. Further, smooth *Brucella *infection of macrophages is inefficient with only 40–60% of cells infected *in vitro *after 1 hour [72]. In contrast, *E. coli *are efficiently engulfed and processed through the classical endosome-lysosome pathway. However, this leads to rapid destruction of the bacteria and MHC class II presentation of antigen [73]. To avoid this destructive pathway, we modified our *E. coli *vector to express invasin from *Yersinia*. This effectively made the vector 80–100% invasive to not only professional phagocytic cells, but to all cells expressing β1-integrin (Table 2, Figure 1). Further, the endocytic pathway was changed as evidenced that live recombinant *E. coli *could be isolated from macrophages after 3 hours (Table 2) whereas wild-type *E. coli *were destroyed. The pathway seemed to mimic that of *Yersinia *as demonstrated by TEM (Figure 2) where the bacterium adheres to a filopodium then is internalized to individual endosomes [74]. The result is more cells internalizing the vaccine with potential to express antigen in association with MHC class I. Of great interest was the fact that *in vivo*, the vaccine expressed the reporter gene (*lux*) for a prolonged period at the site of immunization (Figure 3) as only viable bacteria continue to express lux. This confirms broad cell-type internalization and probable increased antigen presentation.

In addition to *invasin *of *Yersinia pseudotuberculosis *our recombinant *E. coli *vaccine vector co-expressed the *hly *gene of *Listeria monocytogenes *on the same vector. Modification of the bacterial vaccine to express listeriolysin O (LLO) was to increase MHC I presentation of the expressed antigen delivered by the vaccine. As reported by others [51], the bacteria would be lysed in the phagosome/lysosome. Through the pore-forming action of LLO, the cytoplasmic contents of our bacterial vaccine vector (including the over expressed antigen) would then escape into the cytosol and thereby be processed by the proteasome. *In vitro*, this LLO-mediated process has been shown to improve MHC I presentation of antigens by macrophages and dendritic cells [34,35,43,44]. *In vivo*, *E. coli *vaccines expressing LLO induced a very strong anti-tumor CTL response [43]. We did not confirm improved MHC I presentation of GFP antigen by LLO in studies presented here. However, we did see less YFP gene delivery for mammalian cell expression using recombinant *E. coli *without LLO (Table 3; data not shown). Furthermore, a recent report demonstrated that the presence of LLO in a recombinant bacterial vaccine suppresses CD4^+ ^regulatory T cell (Treg) inhibition of antigen-specific CD8^+ ^T cell expansion [51]. Primary immune responses activate antigen induced Tregs limiting vaccine efficacy [75]. The cytokine profile of the primary immune response to our recombinant *E.coli *vaccine vector revealed a mixed Th1/Th2 profile suggesting a high population of CD4^+ ^T cells and possibly Tregs (Table 4). However, the secondary immune response to the vaccine shifted to a Th1 dominant cytokine profile (Table 5) and subsequent generation of antigen specific CTLs (Figures 6 and 7). It would be interesting to determine whether LLO expression in our vaccine vector affected successful CTL generation and long-term CD8^+ ^effector memory T cells.

Three major regulatory cytokines, TNFα, IL12, and IFNγ, were increased in expression relative to controls in both primary immune response (Table 4) and secondary immune response (Table 5) using our recombinant *E. coli *vaccine vector indicating DC maturation and cell mediated immunity. TNFα is a multipotent proinflammatory cytokine fundamental for defense against a variety of intracellular pathogens and is primarily involved in DC maturation [76,77]. DCs infected with *E. coli *clearly show a high capacity to induce the response of naïve T cells, and TNFα secretion by DCs infected with *Brucella *as well as *E. coli *was directly implicated in the maturation of these cells, since TNFα blocking antibodies cause a strong maturation decrease [61]. Invasive *E. coli *vaccine, similar to *Brucella*, initiates the first phase of a T cell dependent adaptive immune response inducing the secretion of IL12 from APCs. IL12 then potently stimulates IFNγ production by activated naïve T cells [78]. Both IL12 and IFNγ are considered essential for protection against brucellosis [10]. Our inclusion of a murine IL12 mammalian expression plasmid in the *E. coli *vaccine vector results in a high level of IL12 expression in the infected cell (Figure 4). This IL12 rich microenvironment surrounding the host antigen presenting cell (professional or non-professional; Table 2) may be involved in supporting the Th1 profile of the secondary immune response as indicated by the high levels of TNFα and IFNγ (Table 5). The resulting maturation of DCs and CD8^+ ^T cells would lead to cell mediated immunity.

The initial host defense to infection is stimulated by pathogen associated molecular patterns (PAMPS) common to different groups of pathogens. The toll-like receptor (TLR) family has emerged as a major group of signaling receptors for PAMPs [79,80]. Classical LPS activates macrophages and DCs through binding the TLR-4. Nevertheless, the respective effects of APC stimulation by isolated LPS or living bacteria are clearly distinct, even when the bacteria carry a highly active LPS like *E. coli*; the bacteria probably bind not only to TLR-4 but also to a set of various receptors. Our studies demonstrate a notable Th1, specific CTL response to antigen delivered by the invasive, recombinant *E. coli *vaccine vector. However, the highly active LPS and PAMPS of *E. coli *may over stimulate the immune response to the vector. Engineering the *E. coli *genome to make the organism less stimulatory to the host would greatly improve the usefulness of this novel vaccine approach.

## Conclusion

We began our studies with the goal of developing a live vaccine vector using an organism (*E. coli*) that was not pathogenic to the host and engineering it to mimic the bacterial pathogen *Brucella *intracellular infection to stimulate a protective cellular immune response. Our data show that this vaccine vector could efficiently infect cells of multiple tissues. These vaccine infected cells acting as antigen presenting cells can stimulate a cellular immune response with Th1 cytokine profile and specific CTLs. Studies are now in progress to determine whether this recombinant invasive *E. coli *vaccine vector, expressing pools of immunodominant *Brucella *antigens, would be sufficient to induce a protective immune response in mice. Our studies show that this novel vaccine could be applied to any disease where cellular immunity is required.

## Competing interests

The authors declare that they have no competing interests.

## Authors' contributions

JH, MD, and DD participated in mouse vaccination studies. JH and DD carried out pentamer staining and cytokine profiling. MD performed IL12 expression studies and flow cytometry. JH performed molecular and cell biology studies engineering and immunoassays. JH and GS conceived of the study, and participated in its design and coordination and helped to draft the manuscript. All authors read and approved the final manuscript.
